# Esthetic Rehabilitation of a Severely Compromised Anterior Area: Combined Periodontal and Restorative Approach

**DOI:** 10.1155/2014/658790

**Published:** 2014-02-13

**Authors:** Rachele Censi, Virna Vavassori, Andrea Enrico Borgonovo, Dino Re

**Affiliations:** ^1^Department of Implantology and Periodontology III, Istituto Stomatologico Italiano, Milan, Italy; ^2^Department of Oral Rehabilitation, School of Oral Surgery, Istituto Stomatologico Italiano, University of Milan, Milan, Italy; ^3^Department of Oral Surgery, Dental Clinic, Fondazione IRCCS Policlinico Ospedale maggiore Ca' Granda, Milan, Italy; ^4^Department of Oral Rehabilitation, Istituto Stomatologico Italiano, Milan, Italy

## Abstract

The complete oral rehabilitation of patients demanding a beautiful and attractive smile involves a multidisciplinary approach that includes the change of both the morphological aspect of the teeth and the architecture of gum tissues. This clinical report describes a successful interdisciplinary approach for the treatment of an esthetically compromised dentition. In a first phase, the periodontal plastic surgery was performed for root coverage and, in particular, it was decided for the execution of a coronally advanced flap for the treatment of multiple recession defects. Once complete healing of soft tissues was obtained, six lithium disilicate veneers were placed over the anterior maxillary teeth. Lithium disilicate is a glass-based ceramic which presents excellent aesthetics and allows the passage of light without creating unnatural reflections. This feature has made it possible to recreate a natural aspect of teeth that in combination with the harmonic architecture of soft tissue has permitted obtaining a beautiful and pleasant smile.

## 1. Introduction

From antiquity to modern times, the face and its expressions have always played a crucial role because they can greatly affect interpersonal relationships [1].

According to several studies, it is especially the smile that influences the appearance of the face as a beautiful smile seems to convey serenity, safety, and success in the beholder [2]. Therefore, patients who turn to dentists require, in addition to the functional aspect, an outcome that meets their esthetic needs in order to obtain a natural smile and beautiful teeth [3]. The professional, in consideration of these demands, can improve the patient's smile in a comprehensive manner, changing both the morphological characteristics of the teeth (shape, color, position, and size) and the architecture of the soft gum tissue [4]. To do this, different techniques of periodontal surgery have been developed for the correction of soft tissue and many changes have been done in the discovery of methods and materials for the construction of direct and indirect restorations.

In this work, we present a clinical case of a patient who required, for cosmetic reasons, the replacement of the previous restoration and the correction of the soft tissue profile at the level of the anterior region of the maxilla. The gingival recessions were treated with a coronally advanced flap, and once healing was complete, prosthetic veneers made of lithium disilicate were placed over the teeth.

## 2. Clinical Case

**Figure 1:**
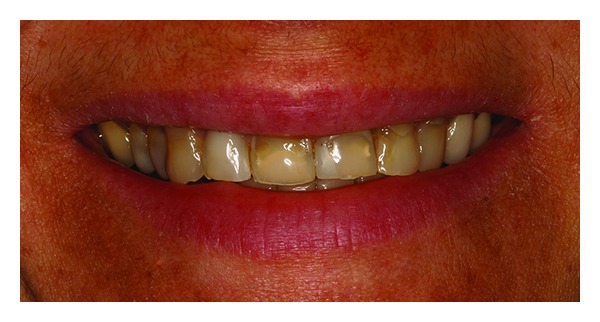
Clinical view of the smile.

**Figure 2:**
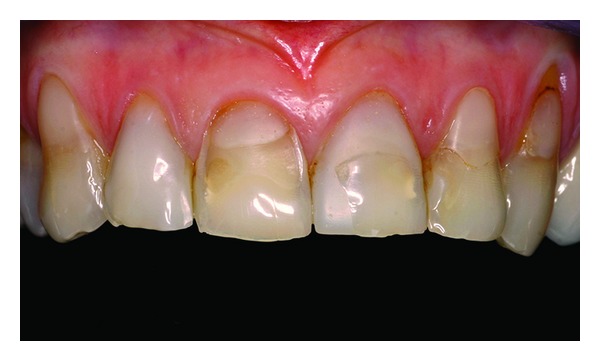
Intraoral clinical aspect.

The patient, C. C., female, aged 45, came to our attention requiring to replace old fillings at the level of the front teeth of the upper jaw and reporting as further blemish the excessive length of teeth with gingival recessions (Figure 1). In a preliminary phase, all the pictures of the case were carried out and, moreover, bitewing radiographs were recorded to assess the presence of any secondary caries (Figure 2). After the clinical-radiographic examination, it was decided to perform the surgery for root coverage in correspondence to the elements between 13 and 23 and to place six prosthetic veneers over the same dental elements once the soft tissue healing was completed. Complete scaling and root planning were performed and oral hygiene instructions were given 4 weeks prior to surgery.

The first question which the clinician has faced concerned the choice of the surgical technique for root coverage. In order to evaluate the better surgical technique suited to the situation, we considered the local anatomical conditions relating to teeth and soft tissues. With regard to the gum tissues, there was evidence that the apical-coronal dimension and thickness of keratinized tissue placed apically to recessions were adequate and, in addition, the vestibule appeared deep enough. Considering the teeth, no deep abrasions were present and no root was displaced buccally. Based on these clinical evaluations, it was decided for the execution of a coronally advanced flap. The choice of this surgical treatment option allows for a greater ease of operation compared to other techniques and a good tolerability by the patient with little postoperative discomfort and, in addition, provides excellent results in terms of root coverage and aesthetics.

**Figure 3:**
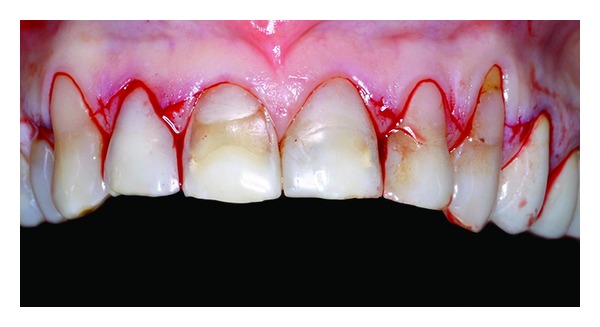
Flap incision.

**Figure 4:**
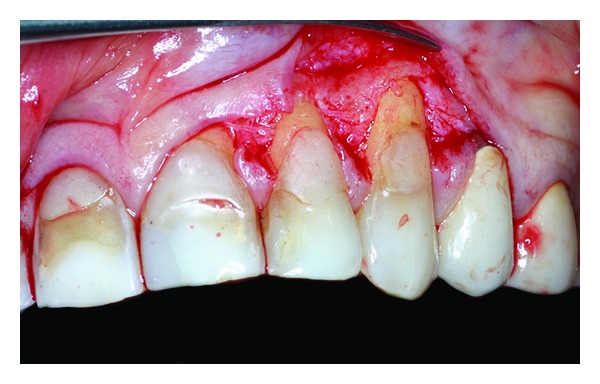
Flap is elevated according to a mixed thickness.

**Figure 5:**
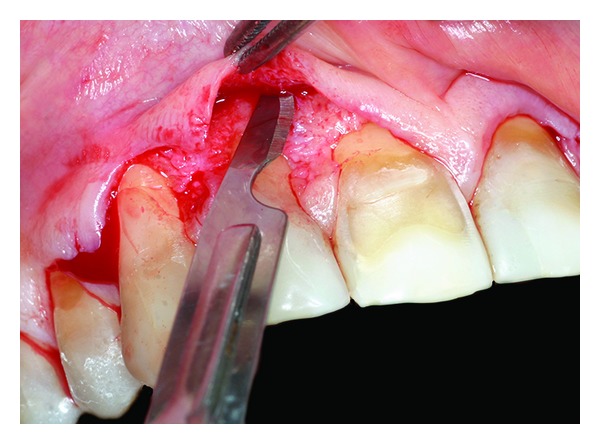
Release of muscle insertions.

**Figure 6:**
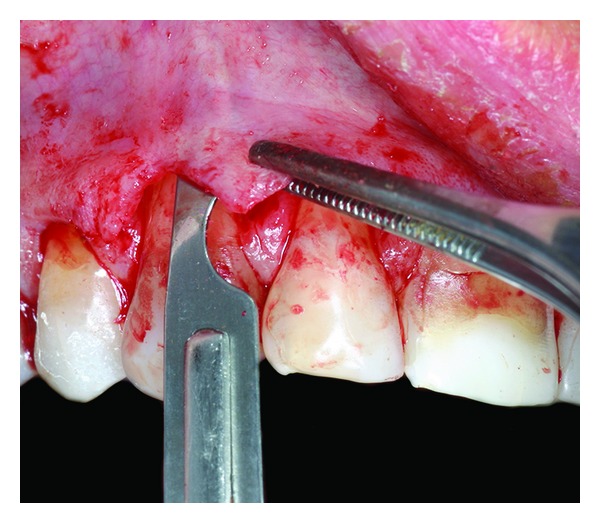
Superficial partial thickness elevation of the flap.

**Figure 7:**
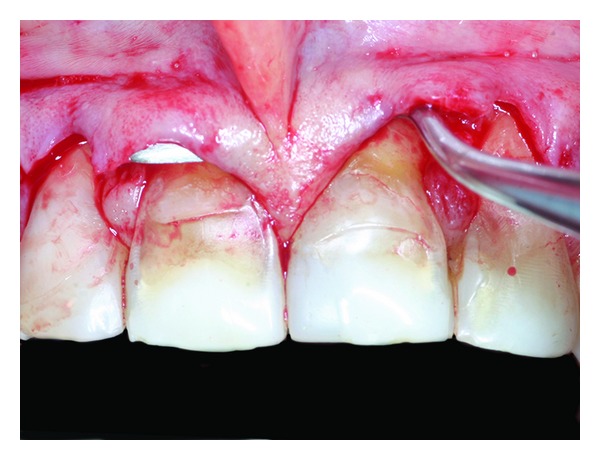
Interincisive papilla is tunneled.

**Figure 8:**
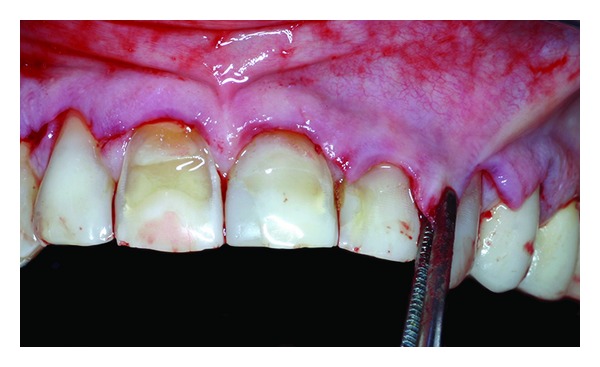
The flap is advanced without any tension.

**Figure 9:**
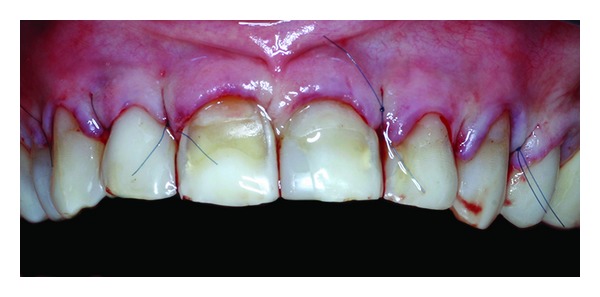
Suture.

For the proposed clinical case, in detail, a coronally advanced flap was done for the treatment of multiple gingival recessions of incisors and canines of the maxillary arch, as proposed by Zucchelli and De Sanctis (2000) [5]. Under local anesthesia (2% mepivacaine with adrenaline 1 : 100.000) the flap was designed with paramarginal oblique incisions in the interproximal areas that were joined together with intrasulcular incision in order to draw the surgical papilla of the flap (Figure 3). The paramarginal incisions were carried out so that all the incisions converged towards the axis of rotation, passing through the center of the interincisive papilla. The flap was elevated according to a mixed thicknesses: partial under the surgical papillas, total apically to the recessions in order to expose 3 mm of buccal bone, and again partial to cut the muscle fibers (Figures 4, 5, and 6). The interincisive papilla was not elevated but “tunnelled,” releasing the muscle insertions below the median frenulum (Figure 7). Once the flap was prepared, the root surfaces were conditioned mechanically with scaler and curettes and anatomical papillae were deepithelialized with a small blade. The flap was coronally advanced to cover the root surface (Figure 8) and subsequently sutured without any tension at the level of the cement-enamel junction (CEJ) (Figure 9). The flap was fixed with a nonresorbable suture material and a mattress sling suturing technique was done. In addition, a U-suture was performed in the alveolar mucosa, in order to reduce the tension of the lips on the edge portion of the flap. The horizontal borders of the suture were included in the surgical area. No periodontal dressing was used.

**Figure 10:**
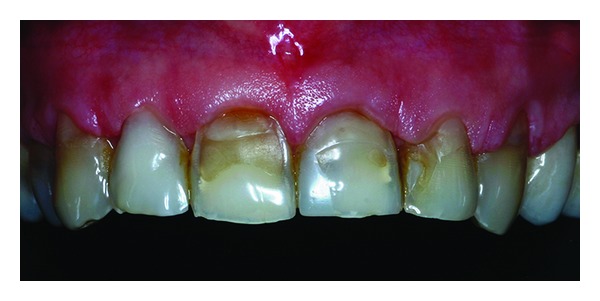
Soft tissue health two weeks after surgery.

**Figure 11:**
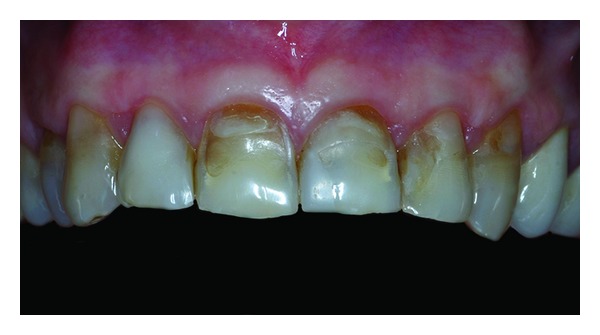
Soft tissue health 3 months after surgery.

All postoperative instructions were provided to the patient. In particular, postoperative care included 1 gr amoxicillin and clavulanic acid every 12 hours for 6 days, ibuprofen 400 mg as needed for pain control, and chlorhexidine gluconate 0.2% twice a day for the first three weeks. Sutures were removed after 10 days. No brushing or flossing was allowed in the operation area for three weeks after surgery. Healing was checked every week for the first month and then every 30 days (Figures 10 and 11).

**Figure 12:**
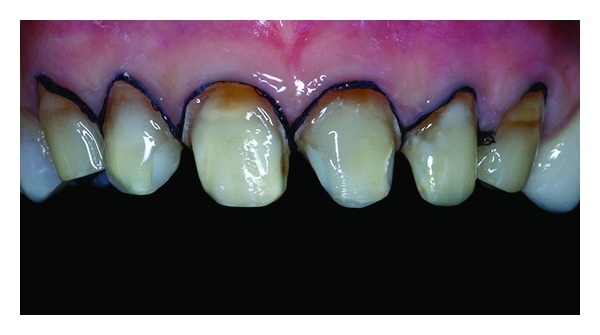
Dental preparation 9 months after periodontal plastic surgery.

**Figure 13:**
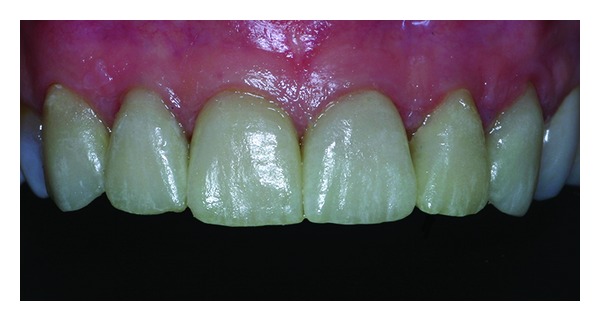
Temporary restorations.

**Figure 14:**
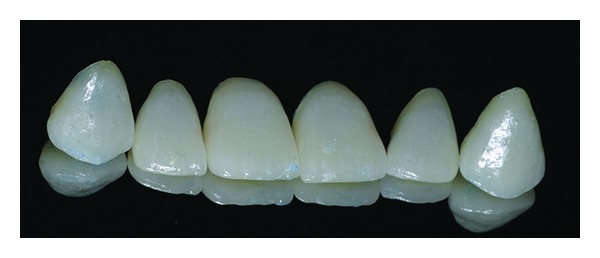
Lithium disilicate veneers.

**Figure 15:**
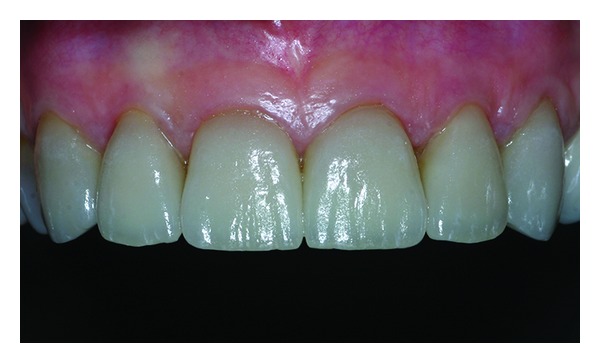
Veneers luting.

**Figure 16:**
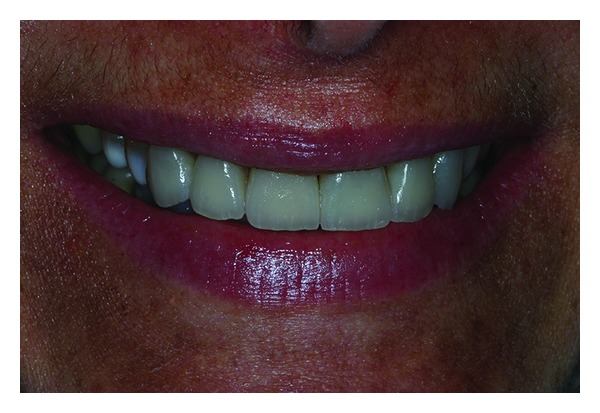
Final clinical result.

**Figure 17:**
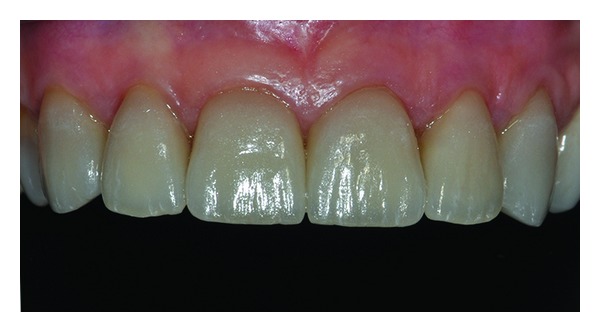
Follow-up 9 months after veneer cementation.

Nine months after surgery, once the complete healing of the soft gum tissue was obtained, the clinician has faced the second clinical question regarding the type of dental rehabilitation. The possible treatment options allow for the execution of direct composite restorations or indirect prosthetic restorations such as veneers or crowns. In this case, the placement of prosthetic crowns would have been a promising treatment aesthetically but overly invasive. For these reasons, on the basis of the clinical evaluation and in order to obtain a successful result in terms of aesthetics and for the long period, it was decided to perform prosthetic veneers. Dental elements were, therefore, prepared and six veneers made of lithium disilicate were adhesively bonded to the surface of the teeth (Figures 12, 13, 14, 15, 16 and 17).

## 3. Discussion

The aesthetics of a smile is determined by the characteristics of the teeth and the harmonious architecture of the gum tissues. However, with regard to the soft tissues, the finding of recessions is not an infrequent event [6]. The gingival recession can be determined by plaque-induced periodontal inflammation and/or trauma during tooth brushing [7]. If a control of these factors is performed, eliminating the etiological factor of inflammation or performing proper tooth brushing, the progression of the recession is avoided. Despite this, the presence of gingival recessions leads to various problems including blemishes, root hypersensitivity, possibility of secondary caries development, and cervical abrasions [6]. The presence of one or more of these factors represents a valid indication for the surgical treatment of the gingival recessions in order to obtain root coverage [8]. The surgical techniques generally used for recession treatment provide different therapeutic options which include pedicle flaps (rotated flaps or advanced flaps), free gingival grafts and subepithelial connective tissue graft.

The choice of the proper surgical technique is essentially based on the evaluation of several factors including the depth and width of the recession defect, the availability of donor tissue, the presence of muscle insertions, and, finally, the patient esthetic needs [5]. The choice of the correct treatment technique is undoubtedly a key point in the treatment plan, but it is important to consider that the treatment success, meaning a complete root coverage (CRC) (CRC is obtained when gingival margin is positioned at the level of the cementoenamel junction (CEJ) or coronally) does not depend on surgical technique, but the soft tissue periodontal support at the level of the interproximal surfaces has an important role in order to achieve successful results. In this regard, Miller in 1985 [9] proposed a classification that relates the type of recession defect with the treatment predictability. This classification separates the recessions into 4 classes and even if replaced by newer ones, it is still the most used.

The periodontal surgery for root coverage is predictable in presence of class I and II defects. Class III defects permit a partial root coverage, whereas it is not possible to get the root coverage for class IV defects.

From the literature, it is noted that the surgical technique which permits obtaining a more predictable and complete root coverage is the bilaminar surgical approach [10, 11]. In this technique, the pedicle flap is associated with a connective tissue graft which requires a second surgical site to harvested the tissue. This approach is inevitably associated with undesirable side effects such as postsurgical pain and discomfort. Considering the coronally advanced flap, additional surgical site in palate is not needed even if this technique achieves excellent results for root coverage, as long as a suitable donor tissue is present [12, 13]. Unfortunately, in the literature, most of the studies refers to the treatment of localized recessions (single tooth) whereas only few studies consider the treatment of multiple recessions. Lindhe and Lang [14] have selected 17 studies in which 527 teeth were treated with coronally advanced flaps and for each case, the percentage of root coverage (PRC) was calculated. The PRC was determined according to the following formula:

(1) %  root  coverage =((preoperative  vertical  recession  depth   − postoperative  vertical  recession  depth)     × (preoperative  vertical  recession)−1)×100.

In this study, the PRC resulted 79%.

The predictability of complete root coverage (CRC) for CAF technique was evaluated in 15 studies in which 287 patients were considered and 499 teeth were treated. It was observed that CRC was of 48% for the treated teeth (if the other surgical techniques are considered, the mean values of CRC were 43% for rotated flaps, 61% for bilaminar surgical approach, and 28% for connective tissue grafts). In these studies, however, there was a very large range of variation (0–90) and, for this reason, it is to possible to declare that these surgical techniques are operator-dependent and probably in many cases the clinicians have not taken into account important factors that influence the final outcome. In general, the surgical techniques for the gingival recession treatment and, in particular, the CAF technique allows obtaining excellent results in terms of root coverage [15]. However, when the correct parables are reestablished at the level of the gingival tissues and the healing of soft tissue is obtained, it is possible to proceed with the rehabilitation of the teeth when the dental elements present shape, size, color, or position that is considered not appropriate. In these cases, the treatment options are direct composite restorations or indirect restorations such as veneers or crowns. The veneers are thin ceramic plates that are cemented on the buccal surface of the front teeth, ensuring optimal aesthetic results [16]. During a preliminary phase, the teeth are prepared but in a minimally invasive way because the tooth preparation is carried out at the level of the enamel. Recently, veneers made of lithium disilicate have been proposed [17]. The lithium disilicate is a glass-based ceramic reinforced with lithium salts. This material has excellent aesthetics and integrates with the tooth in a natural way because it does not present an opaque reinforcement substructure made of gold or zirconium [18]. The disilicate, moreover, is an opalescent material that has the property to allow the passage of light without creating unnatural reflections [19, 20]. This feature makes the use of transparent composite cements for the adhesive cementation of these veneers possible. Besides the excellent aesthetics, the veneers made of disilicate guarantee a resistance up to 3 times greater than the other glass ceramics, because of the presence of lithium salts which give an inherent strength to the structure and, in addition, can be realized with minimum thicknesses (up to 0.3 mm) [21–23]. This characteristic permits the saving of dental tissue.

In the clinical case presented, the application of veneers made of lithium disilicate has allowed the rehabilitation of the teeth with excellent esthetic results whereas the periodontal surgery using the CAF technique has permitted balancing the soft tissue profile obtaining a natural, harmonic, and pleasant smile.
